# Supplementary figures and images for: County-level variations in linkage to care among people newly diagnosed with HIV in South Carolina: A longitudinal analysis from 2010 to 2018
Source: PLoS One. 2023 May 31;18(5):e0286497. doi: 10.1371/journal.pone.0286497 (PMC10231826; doi:10.1371/journal.pone.0286497)

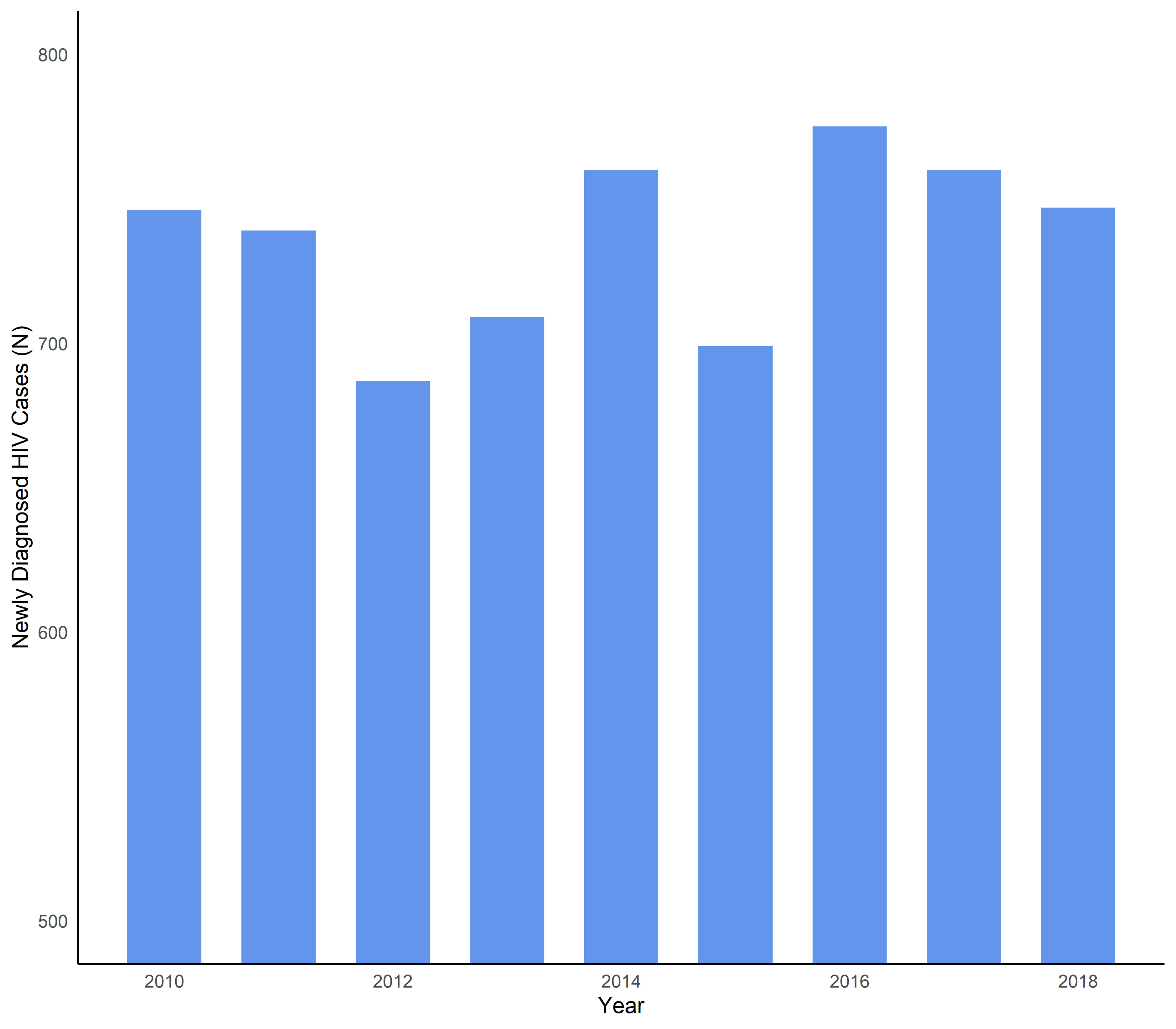

Supplement: S1 Fig — (TIF) [file pone.0286497.s001.tif]

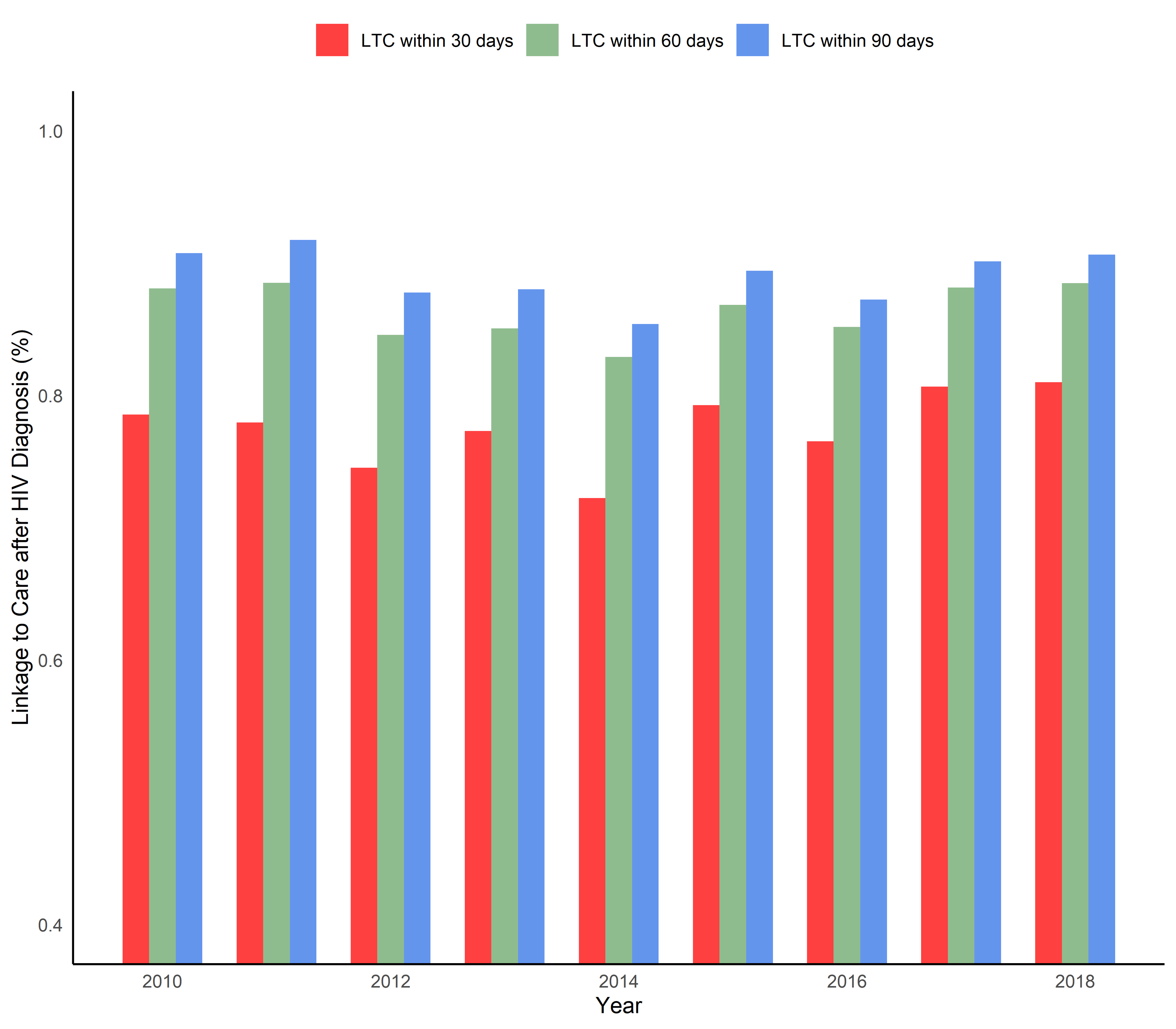

Supplement: S2 Fig — (TIF) [file pone.0286497.s002.tif]

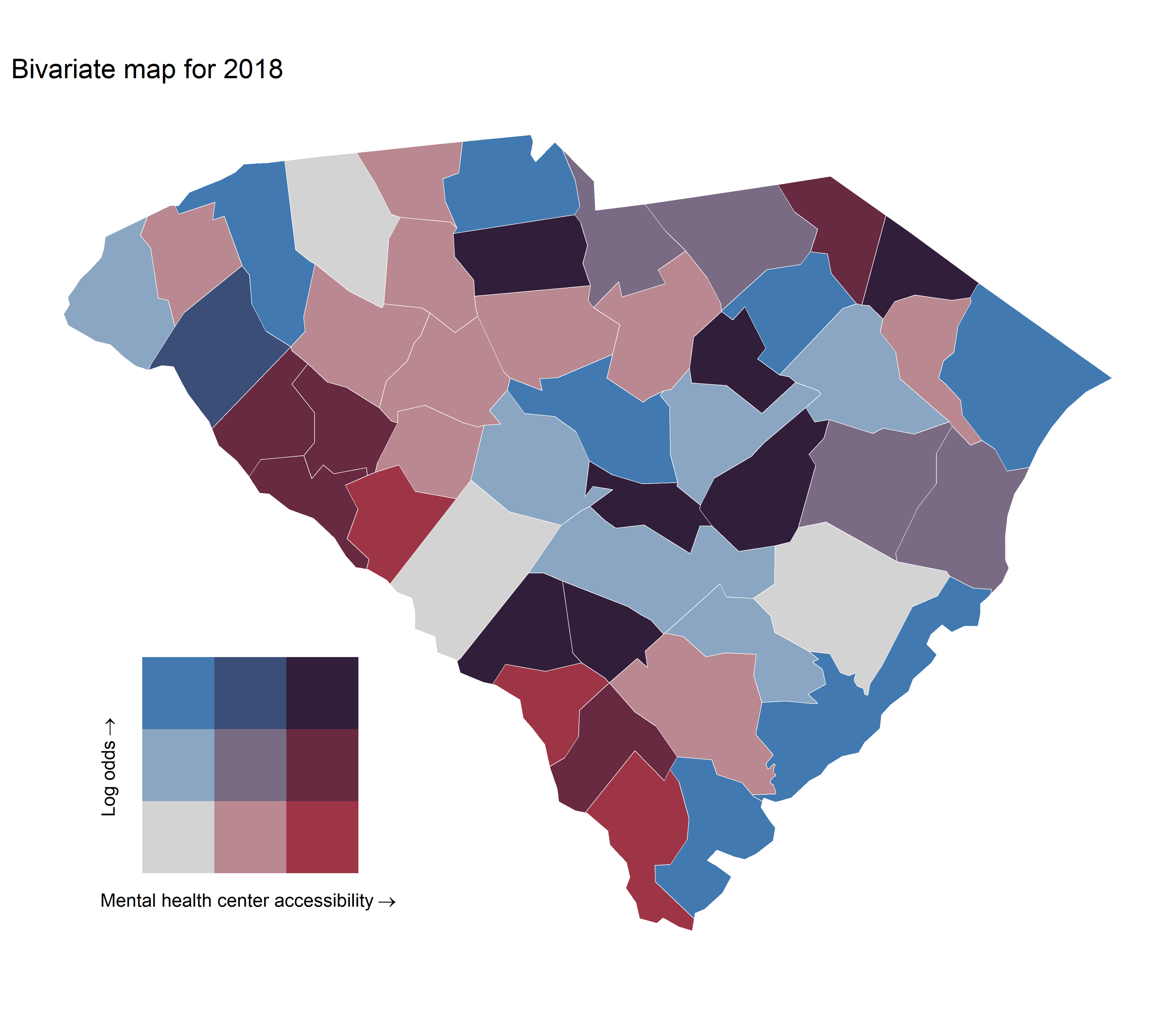

Supplement: S3 Fig — (TIF) [file pone.0286497.s003.tif]
