# Supplementary material for: County-level variations in linkage to care among people newly diagnosed with HIV in South Carolina: A longitudinal analysis from 2010 to 2018
Source: PLoS One. 2023 May 31;18(5):e0286497. doi: 10.1371/journal.pone.0286497 (PMC10231826; doi:10.1371/journal.pone.0286497)
Supplement: S1 Table — (DOCX) [file pone.0286497.s004.docx]

| S1 Table. Jurisdiction(s) meeting National HIV Surveillance System laboratory reporting requirements, 2010–2018 | |
| --- | --- |
| Year | Area of residence meeting National HIV Surveillance System laboratory reporting requirements |
| 2010 | California*, Delaware, District of Columbia, Illinois, Indiana, Lowe, Minnesota, Missouri, Nebraska, New York‡, North Dakota, South Carolina, West Virginia, Wyoming |
| 2011 | California**, Delaware, District of Columbia, Georgia, Hawaii, Illinois, Indiana, Lowe, Louisiana, Michigan, Minnesota, Missouri, Nebraska, New Hampshire, New York, North Dakota, South Carolina, West Virginia, Wyoming |
| 2012 | California*, District of Columbia, Hawaii, Illinois, Indiana, Lowe, Louisiana, Maryland, Michigan, Missouri, New Hampshire, New York, North Dakota, South Carolina, Texas, Utah, West Virginia, Wyoming |
| 2013 | Alabama, Alaska, Arkansas, California*, District of Columbia, Hawaii, Illinois, Indiana, Lowe, Louisiana, Maine, Maryland, Michigan, Missouri, Nebraska, New Hampshire, New York, North Dakota, Oregon, South Carolina, South Dakota, Tennessee, Texas, Utah, Virginia, Washington, West Virginia, Wisconsin |
| 2014 | Alabama, Alaska, California, District of Columbia, Georgia, Hawaii, Illinois, Indiana, Lowe, Louisiana, Maine, Maryland, Massachusetts, Michigan, Minnesota, Mississippi, Missouri, Nebraska, New Hampshire, New Mexico, New York, North Dakota, Oregon, South Carolina, South Dakota, Tennessee, Texas, Utah, Virginia, Washington, West Virginia, Wisconsin, Wyoming |
| 2015 | Alabama, Alaska, California, Colorado, Connecticut, Delaware, District of Columbia, Georgia, Hawaii, Illinois, Indiana, Lowe, Louisiana, Maine, Maryland, Massachusetts, Michigan, Minnesota, Mississippi, Missouri, Montana, Nebraska, New Hampshire, New Mexico, New York, North Dakota, Oregon, Rhode Island, South Carolina, South Dakota, Tennessee, Texas, Utah, Virginia, Washington, West Virginia, Wisconsin, Wyoming |
| 2016 | Alabama, Alaska, California, Colorado, Connecticut, Delaware, District of Columbia, Florida, Georgia, Hawaii, Illinois, Indiana, Lowe, Louisiana, Maine, Maryland, Massachusetts, Michigan, Minnesota, Mississippi, Missouri, Montana, Nebraska, New Hampshire, New Mexico, New York, North Carolina, North Dakota, Oregon, Rhode Island, South Carolina, South Dakota, Tennessee, Texas, Utah, Virginia, Washington, West Virginia, Wisconsin, Wyoming |
| 2017 | Alabama, Alaska, California, Colorado, Connecticut, Delaware, District of Columbia, Florida, Georgia, Hawaii, Illinois, Indiana, Lowe, Louisiana, Maine, Maryland, Massachusetts, Michigan, Minnesota, Mississippi, Missouri, Montana, Nebraska, New Hampshire, New Mexico, New York, North Carolina, North Dakota, Ohio, Oklahoma, Oregon, Rhode Island, South Carolina, South Dakota, Tennessee, Texas, Utah, Virginia, Washington, West Virginia, Wisconsin, Wyoming |
| 2018 | Alabama, Alaska, California, Colorado, Delaware, District of Columbia, Florida, Georgia, Hawaii, Illinois, Indiana, Lowe, Louisiana, Maine, Maryland, Massachusetts, Michigan, Minnesota, Mississippi, Missouri, Montana, Nebraska, Nevada, New Hampshire, New Mexico, New York, North Carolina, North Dakota, Ohio, Oklahoma, Oregon, Pennsylvania¥, Rhode Island, South Carolina, South Dakota, Tennessee, Texas, Utah, Virginia, Washington, West Virginia, Wisconsin, Wyoming |
| Notes: † - Data years 2021 and 2022 have not been assessed for complete lab reporting and may be subject to change. | |
| * San Francisco only; ** San Francisco and Los Angeles only; ‡ New York State (excluding New York City); ¥ Philadelphia only | |
