# Supplementary material for: County-level variations in linkage to care among people newly diagnosed with HIV in South Carolina: A longitudinal analysis from 2010 to 2018
Source: PLoS One. 2023 May 31;18(5):e0286497. doi: 10.1371/journal.pone.0286497 (PMC10231826; doi:10.1371/journal.pone.0286497)
Supplement: Attachment — Submitted filename: Manuscript Review_County-level determinants of linkage to care among people living with HIV in South Carolina.docx [file pone.0286497.s005.docx]

Overall, the authors are bringing to light the social and environment factors that might be contributing to patterns in linkage to care in the state of South Carolina. This is helpful information to learn about, however, there are several items that have me hesitant to accept for publication. Please see below:

1. **Background**
   a. Lines 48 & 52: you state that PLWH and their linkage to care. Linkage to care is not based on persons living with HIV. It is based on persons newly diagnosed with HIV. Throughout the paper, you state PLWH and it should be newly diagnosed. PLWH are based on prevalence. That is, persons who are currently living with diagnosed HIV but could have been diagnosed at any time. The care outcomes for PLWH are receipt of care, retention in care, and viral suppression. <https://www.cdc.gov/hiv/library/reports/hiv-surveillance/vol-27-no-3/index.html>
   b. There are no mention of what HIV looks like in SC. How many cases on average annually? What is the distribution of cases by age, sex, race/ethnicity, transmission category? This information would be helpful to the reader to understand SC.
2. **Methods**
   a. Line 83: eHARS is enhanced not electronic
   b. Lines 57 & 84: How do you calculate LTC rates? If LTC is a percentage? Also, you reference using CDC defn for LTC, you want to cite it here. If it’s the same as reference 4, then it is not clear
   c. Line 86: you state them as “risk factors” however you are obtaining information such as gender, age, race, etc. I recommend not using the word “risk”. Some risk-related terms can be stigmatizing and may imply that the condition is inherent to a person or group rather than the actual causal factors. Could use “variables” or “factors”
   d. What 5-year estimates did you use from the American Community Survey? That is not clear.
   e. For all the data sources, ACS, County Health Rankings & Roadmaps, and US Congress joint Economic Committee, please include what year(s) of data you used.
   f. Why were the variables “Male” and “Black” picked for predictors?
   g. Under the header County-level LTC status (lines 94-103): It appears that percentages, not rates, are calculated. So that readers are clear that your calculation aligns with CDC’s, I highly recommend to use the same language as CDC when reporting on the outcome. Linkage to HIV medical care within 1 month after HIV diagnosis was measured by documentation of  ≥ 1 CD4 (count or percentage) or viral load tests performed ≤ 1 month after HIV diagnosis, including tests performed on the same date as the date of diagnosis <https://www.cdc.gov/hiv/library/reports/hiv-surveillance/vol-27-no-3/content/technical-notes.html>. Also, linkage to care for national-level linkage to care data from CDC include different states for different years (ranging from 14 jurisdictions in 2010 to 43 jurisdictions in 2018 – see Technical Notes from NCHSSTP AtlasPlus here: <https://gis.cdc.gov/grasp/nchhstpatlas/main.html>). Therefore, it may not be accurate to call them national comparisons for the different years (and may need to state which jurisdictions are included in the “national level” for each year you are using).
   h. For Table 1: it would be helpful to include what year(s) for each variable. Also, table 1’s label states, “The detailed definition and cut-point of each county-level predictor”. However, I do not see any cutpoints in the table.
   i. For the ACS data, how did the writer compute data for the 3 years: 2010, 2014, and 2018? Since the 5-year estimate is a period estimate, was the same ACS dataset used for the different years? If so, then that needs to be stated in the methods.
   j. Similar to the previous statement, it needs to be clear which years of each of the 25 variables was used.
   k. Line 164: The statistical analysis section needs to provide more information on exactly how the analysis was computed for each for the 25 variables. Also, it is not clear how the data years (2010, 2014, and 2018) fit into the GEE model. More information is needed for clarity for the reader.
3. **Results**
   a. Lines 177-180: “The state average LTC rate of SC steadily increased from 60.41% in 2010 to 61.27% in 178 2018, but still in a smaller magnitude than the increasing temporal trend at the national level. 179 And this explained why the number of counties with a high LTC decreased from 16 (34.8%) in 180 2010 to 8 (17.4%) in 2018 among 46 counties.” Again, this isn’t an apples-to-apples comparison. There are different numbers of jurisdictions in the national data for each year.
   b. Throughout the results section, please determine if you will use one decimal point or 2. It is not consistent throughout.
   c. Table 3: again, it is not clear if you used the same dataset for the predictor variables for the different years. Also, Ryan White is misspelled in the table (it says Pyan White).
   d. Lines 200-206: There is discussion of counties in different regions (Upstate, Midlands, Pee Dee). This is the first time this information is mentioned. This type of information should be included in the Methods section.
   e. Line 217: The CI should include ~ and not –
   f. Table 4: Some of the confidence intervals have ~ and others have , . Also in Table 4, please include a footnote/note at the bottom of the table stating that the results in bold are statistically significant. Also, what does “N/A” mean? It’s not clearly stated.
   g. There is a concern with some counties having small numbers. The rule of thumb that CDC uses for HIV surveillance data is that results are based on stable numbers (i.e., based on 12 or more diagnoses). If they are less than 12, then they are not considered reliable. It appears there are some counties in your study that run into this issue
   h. Also, there may be some counties with small population sizes. What that taken into account?
   i. For the results, did the writers take into account the number of HIV diagnosis by specific demographics? You results show that males and Black persons were statistically significant findings, but your model didn’t take into account the number of HIV diagnoses that are Black and that are males. From NCHHSTP AtlasPlus, it shows that in just in 2018 alone, here are the numbers and rates for the top 3 racial/ethnic groups:

| **Race** | **Cases** | **Rate per 100,000** | **Population** |
| --- | --- | --- | --- |
| Black/African American | 448 | 40.0 | 1,119,585 |
| White | 172 | 6.1 | 2,815,183 |
| Hispanic/Latino | 56 | 25.9 | 216,019 |

Additionally, there are more males being diagnosed in SC:

| **Sex** | **Cases** | **Rate per 100,000** | **Population** |
| --- | --- | --- | --- |
| Male | 564 | 27.3 | 2,063,390 |
| Female | 161 | 7.2 | 2,232,959 |

Wouldn’t this have some impact on the results? Wouldn’t this introduce some type of bias into your analysis? Also, of those cases diagnosed, what percentage were linked to care for the demographic information? This information would be needed to tell the full picture.

4. **Discussion**
   a. Lines 243-246: For instance, Allendale is a county located in the Lowcountry area with a consistently low LTC rate. This may be because it has the largest proportion of the Black population, the highest percentage of unemployment/poverty, and the lowest median income.” Language that implicitly contains a negative judgement about the character of a person or a group of people (especially the statement about “the Black population”). It also may blame people for circumstances beyond their control. Such language often contributes to disapproving views of, or discrimination against, a group of people. I recommend making sure the statements are written as to not place blame, stigmatize, or offend readers.
   b. For the discussion on counties, there needs to be more understanding of these regions and counties. Readers outside of SC (and even those inside SC), may not know the distribution of population characteristics for the state. Therefore, further explanation is needed. The geospatial work could actually be a paper in and of itself.
   c. Lines 250-256: You found a negative association between % Black and high LTC. Again, couldn’t some of this be explained because most newly diagnosed cases are among Black persons? The study you mention from Florida is a good suggestion for your analysis. Using a multi-level (or HLM) modeling to that take into account both individual and group level factors.
   d. Lines 257-265: Similar to above, your study found a negative association between males and high LTC. Again, couldn’t some of this be explained by most cases being among males?
   e. Lines 286 and 294: the fact that the numbers are so small, these results may be very unstable. There should be caution when presenting results from such case count. Therefore, there should be no discussion on these results.
5. Overall Recommendations
   a. Background needs to be reworked based on recommendations above
   b. Methods needs to include pertinent information that is recommended above
   c. The analysis needs to be reconsidered. Suggestion is to run a multilevel analysis that looks at individual and group level data
   d. Proceed with caution when discussing numbers less than 12
   e. Overall, several concerns with the analysis and results (and interpretation of some of the results)
   f. Be aware of language that is stigmatizing
